# Role of Homocysteine in the Ischemic Stroke and Development of Ischemic Tolerance

**DOI:** 10.3389/fnins.2016.00538

**Published:** 2016-11-23

**Authors:** Ján Lehotský, Barbara Tothová, Maria Kovalská, Dušan Dobrota, Anna Beňová, Dagmar Kalenská, Peter Kaplán

**Affiliations:** ^1^Institute of Medical Biochemistry and BioMed, Jessenius Faculty of Medicine, Comenius University in BratislavaMartin, Slovakia; ^2^Institute of Histology and Embryology, Jessenius Faculty of Medicine, Comenius University in BratislavaMartin, Slovakia

**Keywords:** hyperhomocysteinemia, ischemic preconditioning, intracellular signaling, MAP kinases, neurodegeneration, brain

## Abstract

Homocysteine (Hcy) is a toxic, sulfur-containing intermediate of methionine metabolism. Hyperhomocysteinemia (hHcy), as a consequence of impaired Hcy metabolism or defects in crucial co-factors that participate in its recycling, is assumed as an independent human stroke risk factor. Neural cells are sensitive to prolonged hHcy treatment, because Hcy cannot be metabolized either by the transsulfuration pathway or by the folate/vitamin B12 independent remethylation pathway. Its detrimental effect after ischemia-induced damage includes accumulation of reactive oxygen species (ROS) and posttranslational modifications of proteins via homocysteinylation and thiolation. Ischemic preconditioning (IPC) is an adaptive response of the CNS to sub-lethal ischemia, which elevates tissues tolerance to subsequent ischemia. The main focus of this review is on the recent data on homocysteine metabolism and mechanisms of its neurotoxicity. In this context, the review documents an increased oxidative stress and functional modification of enzymes involved in redox balance in experimentally induced hyperhomocysteinemia. It also gives an interpretation whether hyperhomocysteinemia alone or in combination with IPC affects the ischemia-induced neurodegenerative changes as well as intracellular signaling. Studies document that hHcy alone significantly increased Fluoro-Jade C- and TUNEL-positive cell neurodegeneration in the rat hippocampus as well as in the cortex. IPC, even if combined with hHcy, could still preserve the neuronal tissue from the lethal ischemic effects. This review also describes the changes in the mitogen-activated protein kinase (MAPK) protein pathways following ischemic injury and IPC. These studies provide evidence for the interplay and tight integration between ERK and p38 MAPK signaling mechanisms in response to the hHcy and also in association of hHcy with ischemia/IPC challenge in the rat brain. Further investigations of the protective factors leading to ischemic tolerance and recognition of the co-morbid risk factors would result in development of new avenues for exploration of novel therapeutics against ischemia and stroke.

## Introduction

Many experimental and clinical studies provide evidence that co-morbid disorders are potential risk factors for development of vascular disorders in humans including stroke (Lehotský et al., 2009a; Kwon et al., 2014). At present, there are several known factors elevating the risk of ischemic stroke which include transient ischemic attack (TIA), arterial diseases, atrial fibrillation, improper diet and/or obesity and physical inactivity (Dirnagl et al., 2009). As it has been verified by many studies, even mild hyperhomocysteinemia (hHcy) may increase the risk for clinical manifestations of stroke, probably due to the pleiotropic biochemical properties of homocysteine (Hcy) and its impact on venous and arterial atherosclerotic modifications (Refsum et al., 1998; Steele et al., 2013; Kwon et al., 2014; Petras et al., 2014; Williams et al., 2014). In fact, Hcy suppresses NO production by endothelial cells and platelets and increases generation of reactive oxygen species (ROS) by the release of arachidonic acid from the platelets. It also inhibits glutathione peroxidase and thus stimulates proliferation of endothelial cells (see Petras et al., 2014, for review). In addition, Hcy has been shown to inhibit methyltransferases, to suppress DNA repair and to facilitate apoptosis when accumulated inside the cells. Autooxidation of Hcy metabolites results in H_2_0_2_ accumulation (Boldyrev et al., 2013) and challlenging neurons to Hcy metabolites for longer period leads to necrotic cell death (Ziemińska et al., 2003). Clinical studies suggest that elevated homocysteine level frequently parallels progressive aging as well as neurodegenerative and acute disorders of the CNS, e.g., Alzheimer's disease or Parkinson's disease (Dionisio et al., 2010). Designing appropriate animal models relevant to the clinical conditions of human stroke is an important step for studying the disease ethiology. Until now, only sparse studies have been developed to explore the mutual effect of HCy and ischemic preconditioning (IPC) in animal models of ischemic stroke.

In this paper we summarize current overview on homocysteine conversion steps in the organism and present the genetic and metabolic causes of hyperhomocysteinemia-related neurotoxicity. Based on the results from our laboratory, we also document, in this context, that mutual effect of experimental hyperhomocysteinemia (hHCy) and ischemic insult with or without pre-ischemic challenge can have different outcomes on the extent of neuronal degeneration as well as on the intracellular signaling pathways leading to the preconditioning phenomenon.

### Evolution of ischemic tolerance

The brain is the most sensitive organ to hypoxia or ischemia and many endogenous protective mechanisms have been evolved by nature to protect it against the failure caused by the lack of oxygen and energy substrate supply. These mechanisms can also be artificially induced by various approaches resulting in the protective state known as ischemic tolerance (Dirnagl et al., 2009). Many stressors in acute or chronic paradigms are efficient to suppress subsequent injurious/lethal events in the brain caused by hypoxia/ischemia. Hypoxic or IPC is a widely recognized strategy which eventually leads to the state of ischemic tolerance (for review see Rybnikova and Samoilov, 2015; Wang et al., 2015). It could potentially be used as a preventative measure in high risk individuals or as a precaution against secondary stroke following medical procedures such as aneurysm repair or cardiac surgery (Dirnagl et al., 2009; Lehotský et al., 2009a; Thompson et al., 2013). Initial pre-clinical studies of this preconditioning phenomenon (IPC) relied primarily on brief periods of ischemia or hypoxia known as the IPC stimulus but it was later realized that many other stressors, including pharmacological agents, are also effective. Definitive validation of the protective efficacy of preconditioning agents is still missing. On the other hand, some of these strategies/agents are already adapted in clinical practice and thus the future translational approach seems to be promising. Unfortunately, human stroke is not predictable, but the maneuver of postconditioning has a higher potency to elevate protection/adaptation mechanisms, or as a precaution against stroke recurrence (Danielisova et al., 2014). As in many cases, several evidences suggest that preconditioning is beneficial in the short period after stroke since the brain parenchyma in a longer term manifests alterations or tissue damage which are only postponed (Lehotský et al., 2009a; Thompson et al., 2013). More animal and clinical experiments are required to prove and validate the safety and efficacy of these strategies (Dirnagl et al., 2009). Molecular ethiology of IPC is complex and not yet well understood. However, it was shown to affect many pathways in intracellular signaling including receptor modifications, induction of various kinases, e.g., mitogen activating protein (MAP) and switching on apoptotic mechanisms. Cellular factors included in more clinically relevant postconditioning maneuvers represent inhibition of metalloproteinase 9 (MMP-9) expression, which suppresses the extracellular matrix degradation (Chaturvedi and Kaczmarek, 2014; Turner and Sharp, 2016). Remarkably, ischemic tolerance in the brain can also be activated remotely by application of a tourniquet to one of the limbs (Hu et al., 2012; Liu et al., 2016). Remote preconditioning has even been shown to have beneficial effects in human patients with subarachnoid hemorrhage but further studies are still needed (Dirnagl et al., 2009; Lehotský et al., 2009a; Thompson et al., 2013; Cox-Limpens et al., 2014).

In spite of the high clinical relevance, only a limited number of experimental approaches can be found in the literature to describe the influence of co-morbid hyperhomocysteinemia to ischemic damage in animal models of human stroke. Early experimental data which deal with the effect/attenuation of hyperhomocysteinemia on IPC come from cardiovascular studies (Balakumar et al., 2009; Rohilla et al., 2010; Rana et al., 2015). Experiments documented that probably the high degree of oxidative stress occurred in the hyperhomocysteinemic conditions may be responsible for abolishing/attenuation of the cardioprotective potential of IPC (Balakumar et al., 2009). Although IPC-induced cardioprotection has been documented, the experimental data clearly suggest critical abrogation of the beneficial effects of IPC in metabolic dysregulation caused by hyperhomocysteinemia, probably due to reduced release of calcitonin gene-related peptide (CGRP), the over-expression of glycogen synthase kinase-3β (GSK-3β) and phosphatase and tensin homolog (PTEN), impairment of mito-KATP channels, the consequent opening of mitochondrial permeability transition pore (MPTP) protein kinase C delta, and other mechanisms (Rana et al., 2015).

### Cellular toxicity of homocysteine resulting from its metabolic conversions

**Figure 1:**
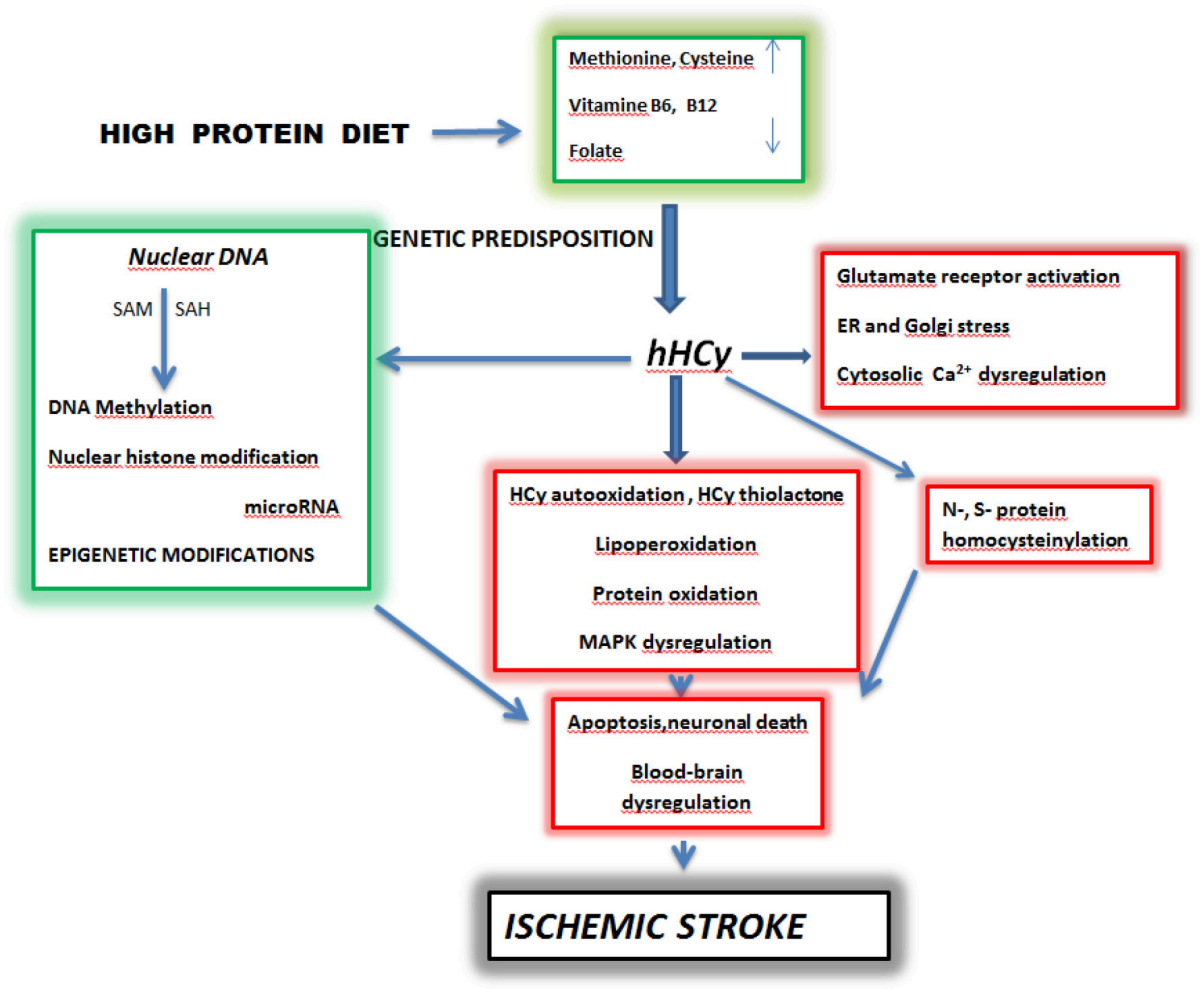
**Schematic pathways of homocysteine toxicity leading to brain ischemic stroke**. SAM-S—Adenosyl Methionine, SAH-S—S -Adenosyl Homocysteine, hHcy—hyperhomocysteinemia. High dietary intake of diet rich in methionine and deficiency of vitamine B_6_, B_12_ and folate lead to hyperhomocysteinemia in predisposed individuals. A prolonged elevated level of homocysteine initiates complex processes which include oxidative stress, protein homocysteinylation and Ca^2+^ dysregulation. These events in parallel with epigenetic changes can culminate in apoptosis, neuronal death and blood-brain barrier dysregulation manifested as ischemic stroke (Kalani et al., 2013; Petras et al., 2014; Kovalska et al., 2015; Lehotsky et al., 2015; Škovierová et al., 2015). Adapted from Lehotsky et al. (2015).

Homocysteine (Hcy) is an intermediate sulfhydryl- containing amino acid derived from methionine. It has been proved to be toxic for neuronal and vascular endothelial cells (Figure 1). In humans it comes from the dietary protein rich in sulfur aminoacids through S-adenosyl methionine conversion (Medina et al., 2001). The causal link between hyperhomocysteinemia and vascular diseases has been described already in the early 1960s (for review see Durand et al., 2001). Remarkably, patients suffering with severe hyperhomocysteinemia (hHcy) manifest typical clinical cardiovascular symptoms as well as neurological disorders, such as cerebral atrophy, dementia and seizures (Obeid and Herrmann, 2006). As shown by many epidemiological observations the association of folate deficiency and hyperhomocysteinemia is frequently correlated with the incidence of vascular diseases and in recent years, also with the incidence of ischemic stroke (Herrmann and Obeid, 2011; Petras et al., 2014).

Homocysteine is metabolized from methionine by three independent alternative pathways:

- re-methylation,
- transmethylation to methionine,
- trans-sulfuration to cysteine.

Though mutations or polymorphisms in the key genes encoding enzymes of Hcy metabolic pathways have been well elucidated in cardiovascular disorders and also in stroke, the epigenetic mechanisms, such as DNA methylation, chromatin remodeling, RNA editing, noncoding RNAs (ncRNAs) and microRNAs (miRNAs) are now involved in the ethiology of stroke (Dirnagl et al., 2009; Kalani et al., 2014). Clinico-genetic observations prove that genetic polymorphisms of the metabolic genes, such as methylentetrahydrofolate reductase (MTHFR), cystathionine β–synthase (CBS), DNA methyltransferase (DNMT) and nicotinamide N-methyl-transferase (NNMT) might be involved in the propensity for stroke due to elevated level of Hcy (Hozyasz et al., 2012; Balcerzyk et al., 2015). Nutritional supplements, e.g., folic acid (a co-factor in one-carbon metabolism), can prospectively interfere with the epigenetic regulations of neuronal cells and may also be involved in the sustainability of neuronal functions and integrity (Obeid and Herrmann, 2006; Kalani et al., 2013).

## Homocysteine metabolic conversions

**Figure 2:**
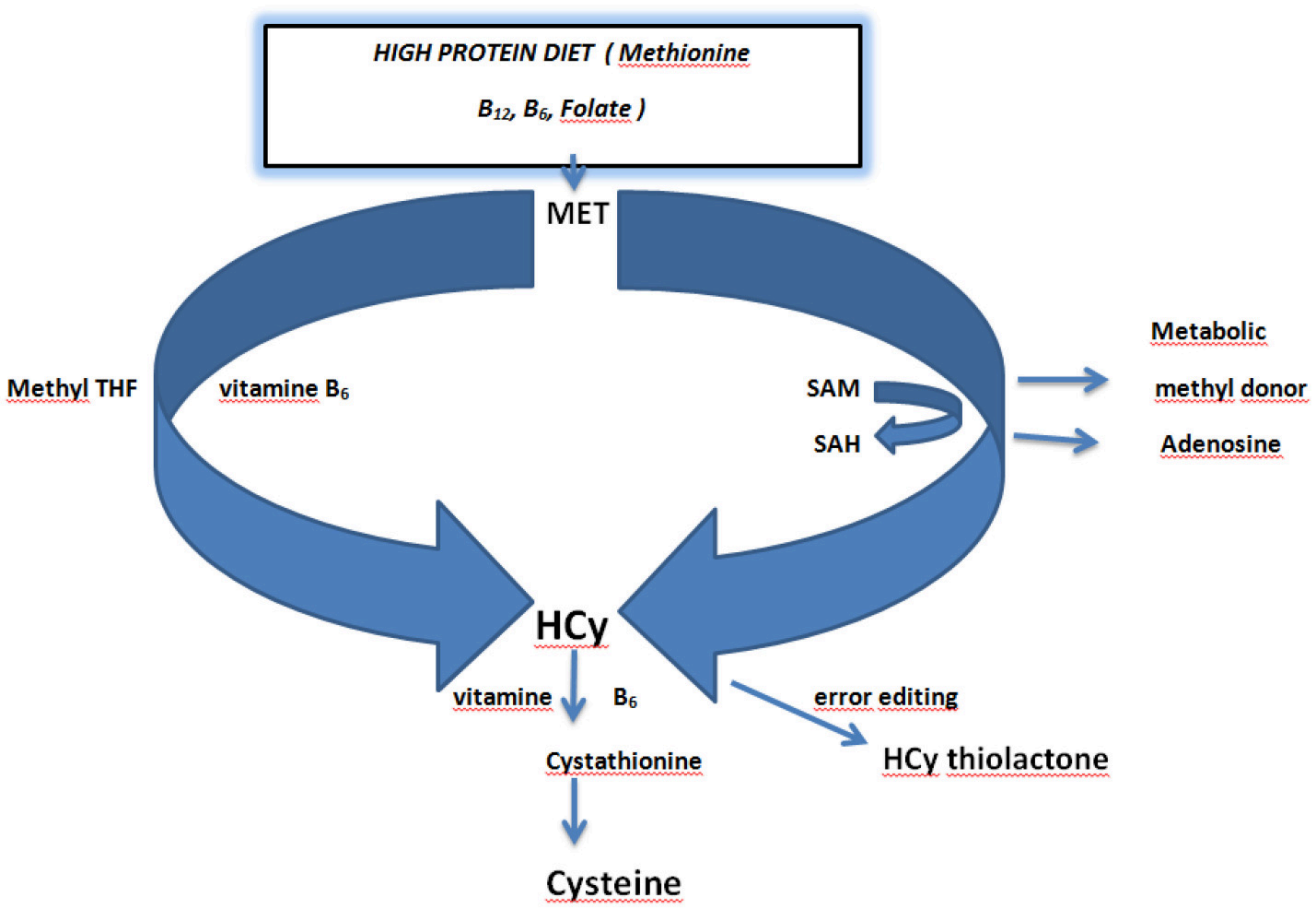
**Schematic overview of homocysteine metabolism and the role of dietary vitamins folate and vitamin B_6_**. Dietary methionine acts as a methyl donor via conversion of S-Adenosyl Methionine (SAM) to S-Adenosyl Homocysteine (SAH). SAH is converted to homocysteine by releasing adenosine. Methionine is directly converted to homocysteine in the presence of Methyl tetrahydrofolic acid (THF) and vitamin B_6_. Homocysteine is converted in error editing reaction to homocysteine thiolactone. Conversion of homocysteine to cysteine requires vitamin B_6_ (Kalani et al., 2013; Petras et al., 2014; Lehotsky et al., 2015). Adapted from Lehotsky et al. (2015).

In humans Hcy is metabolized mainly by methyl group transfer and re-methylation and requires presence of dietary vitamin B_12_ and folic acid for N-5-methyltetrahydrofolate-homocysteine methyltransferase activity. Additionally, the trans-sulfuration reaction of Hcy depends on the presence of dietary vitamin B_6_ (Figure 2). Hcy metabolism in brain parenchyma has some peculiarities in comparison to other organs. The trans-sulfuration pathway is entirely restricted since the remethylation pathway is using betaine (an intermediatory product) (Smulders et al., 2006; Petras et al., 2014) and the possibilities to metabolize/convert HCy mostly depend on the external supplies of folate and cobalamin as dietary vitamins. The glial cells possess very low stores of vitamin B_12_ that are quickly depleted during its negative balance and as such cannot sufficiently support neuronal survival. The toxicity of HCy to CNS neurons is widely recognized affecting both the neuronal survival rate and the ability of neurons to transmit signal and thus to form functional neural networks. This demonstrates that Hcy effects go far beyond the neuronal survival. In our experiments we have evaluated the neurotoxic properties of Hcy on glial cells, using a glioblastoma cell line as a model system. The viability of cells was assayed both biochemically and cytologically. At Hcy concentration around 50 μmol/l in the culture medium (clinically comparable to intermediate hHcy) we observed significant cell death which allowed us to suggest that Hcy-induced impairment of neuronal functions with damage to the glial cells might contribute to the etiopathogenesis of neurological disorders associated with hHcy (Škovierová et al., 2015). Interestingly, the increased Hcy level in humans has been detected both in acute CNS disorders, such as stroke, and chronic diseases such as epilepsy, Alzheimer's disease, dementia, as well as in clinically manifested classical homocystinuria (Seshadri et al., 2002; Kwon et al., 2014; Petras et al., 2014). Very recently it has been reported that the ratio between S-Adenosylmethionine (SAM) and S-Adenosylhomocysteine (SAH) should be used as a biomarker and may provide a sensitive indicator for the clinical diagnosis of atherosclerosis (Zhang et al., 2016). Another scientific group investigating the effect of Hcy on fatty acid binding protein 4 (FABP4) suggested that FABP4 plays a key role in Hcy mediated disturbance of lipid metabolism and that DNA methyltransferase 1 (DNMT1) may be a novel therapeutic target in Hcy-related atherosclerosis (Yang et al., 2015).

## Homocysteine transport in the brain

Little is known about Hcy transport in the brain. Early animal studies indicated that Hcy can be transported through the blood brain barrier (BBB) epithelial cells via a specific saturable receptor in addition to simple diffusion (Grieve et al., 1992). It was demonstrated that in the rat capillary endothelium L-homocysteine shares both the sodium-dependent and independent cysteine transporters (XAG, L, ASC, A) with a varying degree of affinity (Büdy et al., 2006). However, in subjects with moderate hHcy, the majority of Hcy is disulphide-linked to plasma protein cysteine residues, and is, therefore, unavailable for transport via the cysteine transport systems (Sengupta et al., 2001; Lim et al., 2003). Ho et al. (2003) showed an increased production of Hcy in neuronal cells incubated in the folate-deficient media demonstrating that Hcy can be produced within the brain parenchyma. Moreover, accumulation of Hcy in the brain has been shown to be associated with the increased concentration of total plasma Hcy and S-adenosylmethionine (SAM) in the cerebrospinal fluid (CSF) (Herrmann and Obeid, 2011). Kamath et al. (2006) found a significant toxic effect of elevated Hcy on brain microvessels and implicated a role for Hcy in the disruption of the BBB, particularly affecting astrocytes. Thus, Hcy-induced endothelial and astrocytic dysfunction could also alter neuronal function.

## Causes of clinically recognized hyperhomocysteinemia in humans

As in the case of other metabolites, the clinically accepted reference total plasma Hcy concentration varies between 5 and 10 μmol/l. According to the laboratory analyses, classification of hHcy severity depends on its concentration in the plasma: mild (for concentration slightly above 10 μmol/l, moderate (for concentrations between 16 and 30 μmol/l), intermediate (for concentrations of 31–100 μmol/l) and severe (for concentrations higher than 100 μmol/l) which is also manifested with clinical symptoms of homocystinuria (Herrmann and Obeid, 2011). hHcy develops as a result of impaired Hcy metabolism under conditions of severe dietary deficiencies in folic acid, vitamin B_6_ and/or vitamin B_12_ (Figure 1). The hHCy can also be a result of genetic predispositions due to perturbations of genes in methionine and homocysteine metabolism, most likely by methylenetetrahydrofolate reductase (MTHFR) deficiencies. Although Hcy is produced in all tissues, its conversion takes part only in the liver/kidney, mainly through the trans-sulfuration pathway. That is why such tissues as the blood vessels and the brain utilize remethylation as the only alternative. With significant reduction in MTHFR activity in these organs Hcy cannot be remethylated to methionine, hence accumulates in the blood as well as within the nervous system. Interestingly, the role of the MTHFR C677T polymorphism, as a risk factor for ischemic stroke, has recently been established in different laboratories and also in different genetic cohorts (Kim et al., 2013; Song et al., 2016). Remarkably, in orthotopic heart transplantation patients the risk of consequent brain ischemic stroke and transient ischemic attack (TIA) well correlates with plasma Hcy levels. Thus, hHcy could be involved in the pathogenesis of these stroke types mainly by increasing the risk of atrial myocardial fibrillation (Acampa et al., 2016).

## Toxicity of homocysteine to neural cells as a stroke risk factor

Over the years, several theories concerning the toxicity of Hcy have been elaborated. But despite the efforts, none does clearly explain its toxicity. The toxic effect of Hcy on brain tissue is influenced by the absence of two major metabolic routes for Hcy elimination: betaine-mediated conversion of Hcy to Met and transsulfuration of Hcy to Cys. In addition, Hcy acts as an agonist for both groups of glutamate receptors, metabotropic (group I and III) and ionotropic (AMPA) receptors, as well as for N-methyl-D-aspartate receptor (NMDA) (Boldyrev et al., 2013). Overstimulation of these receptors results in an increased level of cytoplasmic calcium, higher production of free radicals and activation of caspases leading to apoptosis (Mattson and Shea, 2003). Not only neuronal cells are exposed to toxic effects of Hcy, but glial cells too (Verkhratsky and Toescu, 2006; Škovierová et al., 2015). The importance of astrocytes for brain homeostasis assisting neurogenesis, determining the micro-architecture of the gray matter and also energy metabolism has been well documented (Verkhratsky and Toescu, 2006). Hcy mediated NMDA receptor induction of neuronal cells could lead to their death due to the transient activation of extracellular signal-regulated kinases, ERK, MAPK, and p38 MAPK (Poddar and Paul, 2013) that is different from the downstream signaling pathways triggered by other NMDA receptor agonists. The Hcy induced glutamate receptor activated neurotoxicity is widely recognized (da Cunha et al., 2012; Kwon et al., 2014). Interestingly, ischemic insults also activate glutamatergic excitotoxicity with the promotion of neuronal death. Additionally, Hcy acts as an inducer of caspase-dependent neuronal apoptosis via several detrimental pathways, such as DNA damage, poly-ADP-ribose polymerase (PARP) dysbalance, and mitochondrial dysregulation by caspase-3 activation (Kruman et al., 2000; Fang et al., 2014; Kamat et al., 2015). Notably, glial-vascular interface communication as a part of BBB is also affected by Hcy (Mattson and Shea, 2003; Verkhratsky and Toescu, 2006; Loureiro et al., 2010; Kalani et al., 2014). As a consequence, elevated levels of Hcy lead to an enhanced excitatory glutamatergic neurotransmission in different brain regions and, as a result, to neuronal damage induced by glutamatergic derived excessive Ca^2+^ influx and generation of ROS.

In humans, the increased level of Ca^2+^ damages mitochondria by collapsing the mitochondrial membrane potential and suppresses ATP production. Furthermore, the consecutive leakage of cytochrome c from mitochondria as well as ROS activate the caspase 3 pathway which leads to DNA fragmentation (Huang et al., 2001; Kwon et al., 2013).

It was proved that Hcy itself is able to induce BBB disruption (Kamath et al., 2006). This disruption can be due to several different processes. Firstly, Hcy induces an imbalance between the activity of MMP-9 and the tissue inhibitor of metalloproteinase 4 (TIMP-4), in the way of increasing MMP-9 and decreasing TIMP-4 activity (Tyagi et al., 2009). Subsequently, MMP-9 interacts with different components of the BBB and leads to disruption of this structure. Secondly, Hcy acts as an excitatory neurotransmitter for (i) γ-aminobutyric acid (GABA) receptors A, which leads to increased vascular permeability and (ii) NMDA receptor (Betzen et al., 2009). The expression of NMDA receptor is not confined to neurons only. Other cells, including endothelial cells from the cerebral tissue, can also express this type of receptor. Free radicals, inducing up-regulation of the NR1 subunit of the NMDA receptor, increase the susceptibility of cerebral endothelial cells to excitatory amino acids, favoring BBB disruption (Betzen et al., 2009; Tyagi et al., 2009).

One of the first hypotheses suggested that ROS and hydrogen peroxide (H_2_O_2_) formed in redox reactions involving the thiol group of Hcy was responsible for the toxicity of this compound. The major drawback of this hypothesis was that Cys (which is a common amino acid) is not a risk factor for vascular diseases, despite its up to 30-fold higher concentration than Hcy (Jakubowski et al., 2009). However, different studies showed that redox reactions may be a key factor in the development of atherosclerosis, vascular hypertrophy and possibly neurotoxicity in animals with hHcy. Thus, a dysbalance in the redox state and oxidative stress can be a primary mechanism responsible for hHcy-related pathogenesis, such as vascular hypertrophy, thrombosis and atherosclerosis (Dayal et al., 2004; Herrmann and Obeid, 2011; Marković et al., 2011; Petras et al., 2014). ROS are generated during oxidation of the free thiol group of Hcy, when Hcy binds via a disulphide bridge with plasma proteins—mainly albumin—or with other low-molecular plasma thiols, or secondarily with another Hcy molecule. The proposed mechanisms for Hcy-induced oxidative stress, therefore, can be classified as follows based on the existing knowledge (Jones et al., 1994; Streck et al., 2002; Tasatargil et al., 2004; Topal et al., 2004; Jiang et al., 2005; Lubos et al., 2007; Loureiro et al., 2010; Jakubowski, 2011; Boldyrev et al., 2013; Petras et al., 2014):

- Restriction of the activity of cellular antioxidant enzymes,
- Hcy auto-oxidation,
- nitric oxide synthase (NOS)-dependent generation of the superoxide anion via uncoupling of endothelial NOS (eNOS),
- disruption of extracellular superoxide dismutase from endothelial surfaces, and
- activation of NADPH oxidases.

Moreover production of a strong oxidant peroxynitrite activates tyrosine nitration and results in modifications of protein function and cellular dysfunction (Postea et al., 2006).

Hcy can also be converted to its thioester forming the Hcy-thiolactone in an error-editing reaction in proteosynthesis (Gurda et al., 2015). Human and experimental animal studies have documented that formation of Hcy-thiolactone, indeed, is involved in the Hcy patho-biology. Its toxicity is based on a reaction which leads to protein N-homocysteinylation through the formation of amine bonds with protein lysine residues (Jakubowski, 2011), which impairs or alters the structural and functional properties of a particular protein.

Several clinical studies have reported that increased plasma Hcy levels may provoke seizures. In agreement with this finding it was suggested that systemic administration of Hcy at high doses provokes convulsive attacks in mice and it was proposed that similar detrimental effects can be manifested in patients suffering from temporal lobe epilepsy (Baldelli et al., 2010). Consequently, the Hcy-derived chemical reactive metabolites are thought to have an important role in Hcy-induced seizures.

High level of plasma Hcy is a recognized risk factor for developing Alzheimer's disease. Li J. G. et al. (2014) have documented that Hcy exacerbates β-amyloid and tau pathology with plaques and tangles, as well as cognitive deficit, in a mouse model of Alzheimer's disease which supports the concept that dietary lifestyle which leads to hHcy can act as a risk factor and actively contribute to the development of the disease.

Many experimental studies from our and other laboratories (Dirnagl et al., 2009; Urban et al., 2009; Łehotsky et al., 2009b; Pavlíková et al., 2009; Danielisova et al., 2014; Stetler et al., 2014) have documented that ischemia/reperfusion injury (IRI) in rats is followed by time-dependent dysbalance in the redox state in the cortex and hippocampus. The ischemic attack also activates alterations at the genetic level resulting in different expression of mRNA and proteins. Results from other laboratories also demonstrate a beneficial effect of preconditioning to the redox balance and gene expression (Stetler et al., 2014). In spite of high clinical relevance, the published data which described complex effect of IRI and endogenously formed Hcy as a recognized risk factor to the ischemic insult are sparse (Kwon et al., 2014).

An experimental approach (Streck et al., 2002; Loureiro et al., 2010; Lehotský et al., 2011; Pavlikova et al., 2011; Kovalska et al., 2012; Kolling et al., 2016) which is modeling development of hHcy by alimentary fortified or intraperitonealy injected Hcy initiated elevation of lipoperoxidative and protein oxidative products in rat hippocampus (Petras et al., 2014). It is well know that Hcy crosses BBB and reaches a peak of its concentration in the cerebrum and parietal cortex between 15 and 60 min after subcutaneous injection. Plasma Hcy concentration in rats treated via this way achieved the levels similar to those found in homocystinuric patients (moderate hHcy). An increased Hcy level is potent enough to induce and to accumulate hydroxyl radicals as the most powerful free radicals with the ability to remove electrons from other molecules including lipids, proteins, carbohydrates and DNA practically in all cellular components (Kolling et al., 2011; da Cunha et al., 2012; Petras et al., 2014). Thus, as seen from literature data (Streck et al., 2002; Loureiro et al., 2010) and also from our results (Lehotský et al., 2011; Pavlikova et al., 2011; Petras et al., 2014; Kovalska et al., 2015; Lehotsky et al., 2015), chronic elevated levels of Hcy manifest high cellular toxicity. The biological consequence of this toxic effect in the form of neuronal degeneration (measured by the number of Fluro Jade C positive- and TUNEL positive cells) (Lehotský et al., 2011; Pavlikova et al., 2011; Kovalska et al., 2012; Lehotsky et al., 2015) and expressed as the ratio of degenerated cells over intact neurons is elevated in the hippocampus of hHcy animals. This index reaches almost the levels which is obtained after ischemic/reperfusion insult (Kovalska et al., 2014).

Earlier studies have also demonstrated that auto-oxidation of Hcy metabolites results in H_2_O_2_ production with consequent induction of necrotic cell death (Ziemińska et al., 2003; Boldyrev et al., 2013). In the study of Ataie et al. (2013), it was shown that direct intracerebroventricular injection of Hcy activates apoptotic cell death in the *substantia nigra* which is consequently followed by typical Parkinson's disease-like behavior in rats. In the clinical conditions, in Alzheimer's patients and patients with mild cognitive impairment, the plasma levels of Hcy correlates with the alterations in the hippocampal volume and disease progression. Remarkably, this effect is not mediated by cerebral amyloid β peptide deposition, or vascular burden, and as such Hcy-induced oxidative dysbalance is the most likely explanation (Kwon et al., 2013; Choe et al., 2014).

In another study Pavlikova et al. (2011), using the hHcy model in rats, have observed significant variations both in the level of mRNA and protein expression for the calcium pump in the secretory pathways (SPCA1. The important role of this protein in normal neural development and migration has been documented in previous studies (Sepúlveda et al., 2008) and SPCA1 reduction was shown to initiate stress of the Golgi apparatus manifested by the changes in membrane structure and redox dysbalance in neurons. Untill now, no literature papers can be found to describe Hcy effect on the expression profile of the Ca^2+^-transport proteins in neuronal cells and the character of transcriptional regulation of the SPCA1 gene is not yet clarified. As shown by Kawada et al. (2005), the transcription factors Sp1 and YY1 may play role in its gene regulation by the cis-enhancing elements in the 5'-untranslated regions.

Another aspect of hHcy is an intracellular Ca^2+^ mobilization and endoplasmic reticulum (ER) stress (Kalani et al., 2013; Petras et al., 2014), which results in development of apoptotic events, endothelial dysfunction and remodeling of the extracellular matrix in brain parenchyma (Li M. H. et al., 2014). Interestingly, Hcy itself, by interfering with the level of S-adenosylmethionine (as a donor of the methyl group), has also been reported to induce modulation of gene expression through epigenetic alteration of the gene methylation status (Dionisio et al., 2010).

Notably, another etiopathogenic processes related to Hcy-induced neurotoxity might involve modifications of protein structure. Protein homocysteinylation includes:

- S-homocysteinylation and
- N-homocysteinylation,

both of which are considered as posttranslational protein modifications. The degree of protein homocysteinylation correlates with the plasma Hcy level (Kolling et al., 2011) and conversion of Hcy to Hcy-thiolactone (Hcy-TL) results in increased protein N- homocysteinylation. As a consequence, homocysteinylation modifies functions of the proteins and elevates the rate of their proteolysis leading to cell damage (Jakubowski, 2004). *In vivo*, Hcy-TL targets and modifies blood albumin, hemoglobin, immunoglobulins, LDL, HDL, transferrin, antitrypsin, and fibrinogen (Jakubowski et al., 2009). Also, Hcy-TL acts as an inhibitor of Na^+^/K^+^-ATP-ase from the cortex, hippocampus, and brain cells of rats, affecting the membrane potential with deleterious effects for neurons (Rasić-Marković et al., 2009). Elevated plasma levels of Hcy-TL and N-homocysteinylated proteins can result either from the genetic defects in Hcy metabolism or a methionine-rich diet (Jakubowski et al., 2009). Gurda et al. (2015) analyzing the changes in gene expression profiles induced by Hcy and its products using microarray technology, real-time quantitative PCR and bioinformatic analysis have identified 47, 113, and 30 different mRNA regulated by N-homocysteinylated proteins, Hcy-TL and Hcy, respectively, and found that each metabolite induced a unique pattern of gene expression. Major molecular pathways affected by Hcy-TL were chromatin organization, “one carbon” metabolism and lipid-related processes while the major pathways of N-homocysteinylated protein and Hcy were blood coagulation, sulfur amino acid metabolism and lipid metabolism. It all indicated that the main diseases related to all three inductors are atherosclerosis, coronary heart disease and stroke.

In addition to this, it was proved that proteins modified by N-homocysteinylation could act as neoantigens, triggering activation of the inflammatory response which is a key component of atherogenesis, atherotrombosis and stroke etiology. Moreover, these neoantigens induce an autoimmune response and the concentration of autoantibodies is higher in some human pathologies (i.e., cerebrovascular disease, renal failure) comparing to normal individuals (Jakubowski, 2011). N-homocysteinylated proteins in the luminal face of vascular endothelial cells are recognized by specific antibodies and this neoantigen-autoantibody interaction leads to the activation of circulating macrophages, which become responsible for repeated vascular endothelium damage. Furthermore, Hcy-TL impairs the ability of the vascular endothelium to regenerate itself by direct inhibition of lysyl oxidase which is responsible for the correct cross-linking of collagen and elastin in the arterial wall (Raposo et al., 2004).

More recently Petras et al. (2014) documented a 57.9% decrease of Mn^2±^ activated superoxide dismutase (Mn-SOD) activity in cortical mitochondria in the hHcy model in rats. This enzyme belongs to the first line of cellular defense against oxidative injury and has been observed to be diminished in the hHcy group compared to the control group. These results are consonant with very recent experiments of Longoni et al. (2016), which also emphasize a high potency of hHcy conditions for putative posttranslational Hcy induced protein modifications. This likely could lead to enzyme(s) homocysteinylation and thiolation which might contribute to the partial enzyme inactivation. Petras et al. (2014), also reported a small increase of catalase (CAT) activity in the hHcy group compared to the control group which is likely to be a response to the increased level of ROS.

Very little is known about the effect of hHcy on the mitochondrial energy transduction system. The results indicate that hHcy reduces mitochondrial respiration, increases activity of the electron transport chain (ETC) complex II, and inhibits the ETC complex IV activity (Chang et al., 2008). In our laboratory we have investigated the effect of hHcy (on rat heart function, activities of the ETC complexes, mitochondrial protein expression and protein oxidative damage (Timkova et al., 2016). Left ventricular developed pressure, as well as maximal rates of contraction and relaxation, were significantly depressed in hHcy rats. Interestingly, hHcy was accompanied by a significant inhibition of ETC complexesII–IV, whereas activity of the complex I was unchanged. The decline in ETC activities was not associated with elevated protein oxidative damage, as indicated by unchanged protein carbonyl, thiol, and dityrosine contents. Moreover, the level of protein adducts with 4-hydroxynonenal was decreased in hHcy rats. Additionally, 2D-gel electrophoresis with matrix-assisted laser desorption/ionization time-of-flight mass spectrometry did not show alterations in the content of inhibited ETC complexes. However, mass spectrometry analyses identified 8 proteins whose expression was significantly increased by hHcy. These proteins are known to play important roles in the cellular stress response, bioenergetics, and redox balance. Altogether, the results of this study (Timkova et al., 2016) suggest that hHCy induced ETC dysfunction is not causally linked with the altered protein expression. Additionally, an altered expression of other mitochondrial proteins suggests an adaptation response to Hcy-induced myocardial injury.

In this context, Kolling et al. (2016) examined the effects of severe hHcy on brain metabolism, and evaluated a possible neuroprotective role of creatine (50 mg/kg body weight) in young rats (6-day-old). In the *amygdala* region Hcy treatment decreased the activities of succinate dehydrogenase and cytochrome c oxidase but did not alter complex II activity. Hcy treatment also increased the number of cells with high mitochondrial mass, high mitochondrial membrane potential and undergoing late apoptosis. Importantly, creatine administration prevented some of the key effects of Hcy in the amygdala. These authors also observed a decrease in the activity and immuno- content of the α1 subunit of the Na^+^,K^+^-ATPase in the amygdala after Hcy-treatment. These findings support the notion that Hcy modulates mitochondrial function and bioenergetics in the brain, as well as Na^+^,K^+^-ATPase activity and suggest that creatine might represent an effective adjuvant to protect against the effects of high Hcy plasma levels.

Similarly, results from hHcy experiments (Longoni et al., 2016) pointed out the neuroprotective effect of vitamin D (calcitriol) against hHCy as an important factor of brain development, brain metabolism and neuroprotection. The authors showed that pre-treatment with calcitriol up to 250 nmol/l has a remarkable protective effect on cortical slices. Hcy caused changes in bioenergetics parameters (e.g., respiratory chain enzymes) and mitochondrial functions by inducing changes in mitochondrial mass and swelling. Additionally, Hcy induced an increase in NeuN^(+)^/PI cells but did not induce GFAP^(+)^/PI cells. Calcitriol prevented these alterations likely by increasing the level of the vitamin D receptor. These findings suggest that calcitriol treatment may be one of the therapeutic strategies against cerebral complications caused by Hcy.

## Effect of hyperhomocysteinemia on ischemic/reperfusion injury and ischemic tolerance induced by ischemic preconditioning

### Effect of hyperhomocysteinemia on ischemic reperfusion injury (IRI)

Clinical relevance of elevated total HCy level in plasma in the development of human stroke (Shi et al., 2015), stroke reoccurrence and in the prediction of mortality, especially in stroke patients with the large-vessel atherosclerosis subtype (Kumral et al., 2016) has been proved by many studies. Moreover, hHcy contributes in humans to vascular dysregulation, cognitive impairment and dementia (Hainsworth et al., 2016). Increased level of Hcy also predicts the risk of incident dementia which is independent of cerebral small-vessel diseases and vascular risk factors (Miwa et al., 2015).

Interestingly, in spite of the high clinical significance, only a limited number of experimental approaches can be found in the literature to describe the mutual influence of co-morbid hHcy to ischemic damage in animal models of human stroke (Sato et al., 1998; Thompson et al., 2013; Stetler et al., 2014). As shown by several studies, the ischemic/reperfusion insult induces degeneration of the majority (more than 64%) of hippocampal neurons (Dirnagl et al., 2009; Kovalska et al., 2012). An experimental approach (Streck et al., 2002; Loureiro et al., 2010; Lehotský et al., 2011; Pavlikova et al., 2011; Kovalska et al., 2012; Kolling et al., 2016) which is modeling development of hHcy by alimentary fortified or intraperitonealy injected Hcy has proved that in the rat cortical and limbic areas neurons demonstrate functional and morphological changes. The changes lead to the impairment of ion transport mechanisms, mitochondrial alterations and cytoskeltal remodeling. However, by combination of this metabolic stressor with 15 min forebrain ischemia/reperfusion (Kovalska et al., 2012, 2015), morphological changes in the neurons and disturbances of glial cells are aggravated and the extent of intact –like cells is altered in comparison to the naive ischemic/reperfusion group.

The effect of hHcy on cellular degeneration and morphological changes was manifested in rat hippocampal and cortical regions (Kovalska et al., 2012, 2015). The increased number of Fluoro-Jade C+ and TUNEL+ neuronal and glial elements supports toxic effect of hHcy. The thickened and collapsed processes that poorly extend to the area of pyramidal neurons in the CA1 region and M1 cortex are presumably due to the morphological alterations of astrocytes and cytoskeletal remodeling. This might be an indicator of severity of neuronal injury induced by hHCy (da Cunha et al., 2012; Kwon et al., 2014). Astrocytes are highly plastic cells and their dynamic morphological changes could affect the intercellular communication with surrounding synapses that are important in the development of brain lesions (Buffo et al., 2010). Maler et al. (2003) reported that Hcy level of 2 mmol/l and above induced a dose-dependent cytotoxic effect on cortical astrocytes. Astrocytes regulate expression of the NMDA receptor subtypes, which increase neuronal sensitivity to glutamate toxicity and thus accelerate the initial step in the program of reactive astrogliosis and dynamics of astrocyte response to the damage (Škovierová et al., 2015). On the other hand, in response to the injury, astrocytes synthesize a number of factors that may play either neuroprotective or neurotoxic roles.

Increased neuronal damage by Hcy was also detected in *in vitro* ischemic model of hippocampal slices with oxygen and glucose deprivation. Injury of the neural cells was analyzed by quantification of lactate dehydrogenase (LDH) release into the extracellular fluid. Hcy increased the LDH release suggesting an aggravated tissue injury caused by hypoxic/ischemic conditions (Tagliari et al., 2006).

Remarkably, combination of both stressors (ischemia + Hcy) initiated morphological degenerative alteration and also affected considerably the expression of the MAPKs pathways by massive activation of MAPK/p-p38 with a maximum at 24 h after reperfusion (Kovalska et al., 2012, 2015). This dynamic of MAPK/p38 activation could contribute to a more extensive progression of tissue injury (Poddar and Paul, 2013; Zhou et al., 2015). MAPK/ERK are versatile protein kinases that are ubiquitously expressed in the CNS. The studies document the robust expression changes in the hippocampus and modest posttranslational changes in the MAPK/ERK pathway in less sensitive vulnerable neurons of the cortical layers III and V (Zhang et al., 2009; Kovalska et al., 2012, 2015). Extensive experiments have shown an interplay and tight integration of MAPK/ERK signaling in promoting neuronal cell death both in development and in neurodegenerative disorders (Zhang et al., 2009; Poddar and Paul, 2013). It has been proposed that transient activation of MAPK/ERK kinase has different consequences as compared with sustained activation. Transient activation of MAPK/ERK plays a pivotal role in neuronal maturation, survival, and long-term potentiation (Zhou et al., 2015). On the other hand, sustained activation of MAPK/ERK may play a critical role in triggering pro-apoptotic signals and neuronal cell death (Zhang et al., 2009; Poddar and Paul, 2013). It is well known that hHcy mediates glutamate-mediated NMDA receptor stimulation, which eventually leads to the activation of both stimulatory and inhibitory pathways involved in the modulation of MAPK/ERK signaling (Zhang et al., 2009). In fact, the dual role of MAKP/ERK kinases in cell survival and death suggests that a unique profile of gene expression may be elicited depending on the duration and/or magnitude of MAPK/ERK kinase activation (Zhou et al., 2015). Thus, the duration of MAPK/ERK kinase activation following MAPK/p38 stimulation depends on the nature of the extracellular stimuli (in our experimental conditions hHCy and or ischemia or combination of both, Kovalska et al., 2015) and may have different consequences on intracellular signaling pathways eventually leading to different cellular responses. This is in line with previous experiments which documented, that Hcy promotes p38-dependent chemotaxis in bovine aortic smooth muscle cells and this mechanism is important for Hcy-induced atherogenesis with the potential therapeutic implications (Akasaka et al., 2005).

In the context of verification of HCy toxicity, study of Sato et al. (1998) documents that S-adenosyl-L-methionine (SAM) manifested a dose-dependent protection of hippocampal CA1 neurons after transient forebrain ischemia in rats. As was expected, this protective effect was suppressed by S-adenosyl-L-homocysteine challenge, which acts as a potent inhibitor of transmethylation. From these results, authors concluded that the enhancement of cerebral SAM level and activation of transmethylation using SAM as a methyl donor in postischemic brain is vital for protecting neurons against delayed neuronal death. Sharma et al. (2015) proposed that pathological consequences of N-homocysteinylation due to the elevated level of Hcy result in cytotoxicity and even amylod formation. In fact, high levels of Hcy was shown to aggravates cortical neuronal cell injury after cerebral ischemia through neuronal autophagy over-activation detected by significantly increased formation of autophagosomes and the expression of LC3B and Beclin-1and causes increased cerebrovascular permeability (Tyagi et al., 2012; Zhao et al., 2016). As was proposed by authors, the oxidative damage-mediated autophagy may be a molecular mechanism underlying neuronal cell toxicity of elevated Hcy level during ischemic insult.

Interestingly, in another study by Veeranki et al. (2015a) in CBS-/+ mice under the hHcy condition, the satellite cells from the skeletal muscle not only expressed lower *in vitro* proliferative activity, but also manifested increased oxidative stress. In addition, they shown elevation of p38 MAPK as well as p16 and p21 expression after hHcy treatment suggested that hHcy-induced suppression of satellite cell proliferation involves p38 MAPK signaling.

In fact, Tyagi et al. (2012) found homocysteinylated cytochrome-c mediated autophagy in hHcy mice after cerebral ischemia. Cytochrome-c transports electrons and facilitates bioenergetics in the system and authors found that tetrahydrocurcumine ameliorates autophagy during this condition by reducing homocysteinylation of cytochrome-c in-part by MMP-9 activation.

In a clinical settings, Hcy reduces number of endothelial progenitor cells (EPCs) in patients with cerebral stroke through apoptosis. Ischemic insults activates migration of EPCs from bone marrow to repair damaged sites either through direct incorporation of EPCs or by repopulating mature endothelial cells. Increased level of Hcy mediates toxicity to EPC due to apoptosis involving caspase-8, cytochrome c release, and caspase-3 activation and thus making reduction of EPC numbers (Alam et al., 2009). Yang et al. (2016) presented data which indicates that co- morbid Hcy in cerebral stroke stimulates hypermethylation of the trombomodulin gene which leads to gene silencing. Since thrombomodulin may be protective against cerebral ischemia by downregulating coagulation, increased Hcy may play an important role in the occurrence and development of cerebral infarction after ischemic stroke. In a recent paper Caldeira et al. (2014) discusses the role of the ubiquitin-proteasome system in brain ischemia which emphasizes the deletarious role of increased Hcy in the control of ubiquitin-containing proteinaceous deposits accumulation and modulation within the ischemic injury. Furthermore, Toda and Okamura (2016) attributed the idea that hHcy due to the impaired synthesis of NO in the endothelium and by the increased production of asymmetric dimethylarginine is responsible for the impaired circulation in the brain and hypoperfusion/transient ischemia may act as a triggering factor for dementia and Alzheimer's disease. Reduced actions of NO and brain hypoperfusion trigger increased production of amyloid-β that inhibits endothelial function, thus establishing a vicious cycle for impairing brain circulation.

### Combination of hyperhomocysteinemia with preischemia/preconditioning followed by ischemic insult

The pre-ischemic maneuver as a form of evolved tolerance to consequent ischemia is known to rescue the majority of hippocampal neurons (Lehotský et al., 2009a; Kovalska et al., 2012; Rybnikova and Samoilov, 2015; Wang et al., 2015). It has been previously shown that ischemic/reperfusion injury (IRI)t leads to neurodegeneration of neurons in the CA1 region of hippocampus as detected by 75–80% Fluoro-Jade C+ and 90 times (9 ± 1.2 cells/mm2) higher TUNEL+ cells in comparison to the control. On the other side, ischemic precondtioning (IPC), remarkably, leads to suppression of the number of positive cells to more than 70% and conferred neuroprotection (Kovalska et al., 2012, 2014, 2015). Combination of hHcy with ischemic injury increases the extent of neurodegeneration, however, if hHcy is combined with preconditioning (Lehotský et al., 2011; Pavlikova et al., 2011; Kovalska et al., 2012, 2015; Petras et al., 2014; Lehotsky et al., 2015), this maneuver leads to the massive suppression of cell degeneration (as compared to IPC alone) not exceeding 5% of the total number of neurons. It apparently seems that, at least in this hHcy model in rats (Kovalska et al., 2015), IPC significantly corroborates the protective mechanisms.

Recently, we have shown that IPC prior to the lethal ischemia affects MAPK/ERK and MAPK/p38 pathways in the cerebral cortex (Kovalska et al., 2012) as well as in the hippocampus (Kovalska et al., 2014). There is only sparse literature data focusing on the effect of Hcy on the protein expression of MAPKs in neuronal cells (Poddar and Paul, 2013). Poddar and Paul (2013) showed a biphasic response of MAPK/p38 activation in the Hcy-NMDA induced neuronal damage *in vitro*, characterized by an initial rapid elevation followed by a delayed and more prolonged secondary increase, where the later peak was primarily involved in mediating the Hcy-induced cell death. They also showed that this secondary activation of MAPK/p38 correlates with upstream MAPK/ERK activation, which plays a role in facilitating the Hcy-induced cell death. These results are consistent with the conclusions of the study of Kovalska et al. (2014, 2015). In the previous reports from this group (Kovalska et al., 2012, 2014; Lehotsky et al., 2015) it has been shown that IRI induces only a slight increase of MAPK/p38 expression. However, the combination of both stressors (ischemia+ hHcy) leads to the massive activation of MAPK/p-p38 with a maximum at 24 h after reperfusion (Kovalska et al., 2014). This dynamic MAPK/p38 activation could contribute to a more extensive progression of tissue injury (Poddar and Paul, 2013; Zhou et al., 2015). Remarkably, the protective effect of the IPC maneuver detected by the decreased numbers of degenerated neurons is also followed by the decreasing of MAPK/p-p38 expression and elevated immun-osignal for MAPK/p-ERK in ongoing reperfusion times in the CA1 hippocampal region and M1 cortical area.

Significant reductions of oxidative changes and suppression of cell degeneration in the hippocampus as well as in cortex of rat brain is generally an indicative consequence of preischemic treatment (Dirnagl et al., 2009; Lehotsky et al., 2015). Remarkably, IPC in the both paradigms (without and in hHCy condition) preserved the majority of neurons, as seen by decreasing the number of Fluoro-Jade C+ and TUNEL+ neuronal cells. Additionally, preischemia/preconditioning had a protective/stimulatory influence on the MAPK/ERK protein activation under both stress conditions with the opposing effect of MAPK/ERK and MAPK/p38 on cell survival and cell death. Conclusively, preconditioning even if combined with hHcy could still preserve the neuronal tissue from the lethal ischemic effect.

In another set of experiments, we have shown earlier that pre-ischemic treatment prior IRI retains the integrity of the majority of hippocampal neurons and also stimulates recovery of the expression rate of both the SPCA1 mRNA and protein in rats (Pavlíková et al., 2009; Stetler et al., 2014). Combination of hHcy with IRI leads to the slensing of the SPCA mRNA expression and IPC re- stimulates its rate to 2.5-fold.

In conclusion, results from our and other laboratories show that hHcy is associated with a selective degeneration of cortical and limbic structures including the hippocampal area. This degeneration involves the loss of neurons, glial activation, hypertrophy of astrocytes and probably sprouting of new connections. The morphological findings indicate that astrocytes are the first neural cells participating in the deleterious actions of Hcy on the CNS. Apparently, astrocytes are able to respond to mild hHcy by reorganizing their cytoskeleton, surviving and protecting neurons from the damage (Loureiro et al., 2010).

The biological effect of various preconditioning agents is well documented (Dirnagl et al., 2009; Lehotský et al., 2009a). Various types of preconditiong is able to attenuate adverse effect of injurious agents, such as Hcy. As it is shown by the study of Blaise et al. (2009), short hypoxia could suppress the deleterious effects of hHcy on developing rat brain by inducing neurogenesis. Brief neonatal hypoxia, as form of preconditioning, markedly stimulated migration of new neurons to the permissive areas such as the subventricular zone and the hippocampus, increased locomotor coordination and learning and memory and attenuated the long-term effects of hHcy. Similarly, physical exercise (Hrncic et al., 2014) as a type of preconditioned impulse has a beneficial effect in the hHcy induced seizures. It decreases susceptibility to seizures which, according to the authors, is, at least in part, a consequence of improved antioxidant enzymes activity.

Moreover, physical exercise reverses glutamate uptake and oxidative stress effects of hronic Hcy administration in the rats (da Cunha et al., 2012). Wei et al. (2014) documented that application of a chemical metabolic preconditioner hydrogen sulfide inhibits Hcy-induced endoplasmic reticulum stress and neuronal apoptosis in rat hippocampus probably due to the upregulation of the BDNF-TrkB pathway.

In parallel to the above described hHcy induced alterations in intracellular signaling, deleterious effect of Hcy can include also newly described epigenetic dysregulation. The experimental evidence suggests that epigenetic mechanisms are also involved in the etiopathogenesis of stroke and the phenomenon of ischemic tolerance. In particular, it was shown that several important enzymes regulating DNA methylation (e.g., DNA-N-methyltransferas) are implicated in these processes. As a result, its might lead to hypermethylation of the genomic DNA and silencing of functional genes (Kalani et al., 2013).

However, in the context of methylation processes, the one carbon unit metabolism pathway as a part of regular Hcy metabolism correlates with amino acids methylation of the functional proteins and histones as well as of the nucleotides within the RNA and DNA. Remarkably, demethylation of S-adenosyl methionine which gives rise to S-adenosyl-homocysteine, is the sole source of de novo methyl groups for the cell. One can only speculate, that in the hHCy conditions, dysregulation of this step might have a broader implication for many cellular processes including modulation of diverse genes expression via epigenetic regulation (Dionisio et al., 2010; Kalani et al., 2013, 2014). In line with these findings it seems reasonable to consider the association of hHCy and BBB integrity in brain ischemic stroke. As was shown by the recent paper of Kalani et al. (2014), this process could be regulated by epigenetic mechanisms including expression of the specific miRNA29b. This regulates activity of methyl transferase DNMT3b which finally leads to MMP9 activated digestion of the extracellular matrix and junction proteins leading to a leaky vasculature. Moreover, Hcy itself directly affects the BBB permeability and the miR29b regulated activity of MMP9 seems a novel epigenetic mechanism. Similarly, an epigenetic regulation stimulated by hydrogen sulfide is included also in the attenuation process of Hcy-induced mitochondrial toxicity in mouse brain endothelial (bEnd3) cells (Kamat et al., 2015). In this context, the effect of hHcy was investigated in the model of skeletal muscle weakness and fatigability (Veeranki et al., 2015b). Mice with a haplotype of metabolic enzyme CBS+/- exhibited more fatigability and generated less contraction force due to reduced ATP levels. In parallel an increase in the levels of miR-31, and miR-494 that were implicated in dystrophin and mitochondrial regulation and an increase in DNMT3a and DNMT3b proteins and global DNA methylation levels suggest that hHcy plays a causal role in enhanced fatigability through mitochondrial dysfunction which involves epigenetic changes.

Recently has been shown that elimination of the toxic Hcy metabolite, Hcy-thiolactone is performed by the high density lipoprotein (HDL)—associated enzyme, Hcy –thiolactonase/paraoxonase 1 (PON1) (Domagała et al., 2006). This brings a novel pathobiochemical relevance, because it has been suggested that PON1 can protect mice against Hcy-thiolactone neurotoxicity by its hydrolyzing activity also in the brain (Borowczyk et al., 2012).

**Figure 3:**
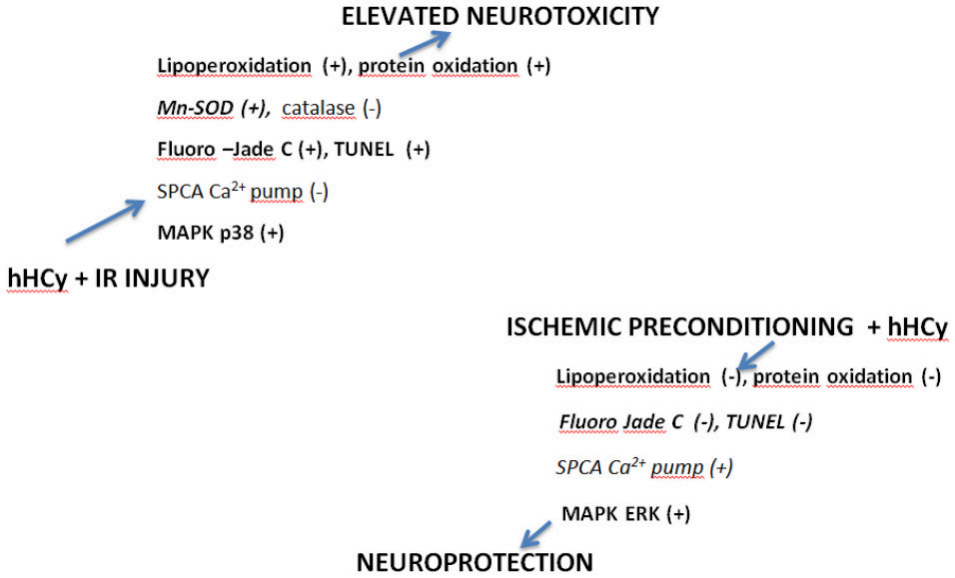
**Proposed mechanisms leading to homocysteine neurotoxicity and the protection induced by ischemic preconditioning (IPC) in hyperhomocysteinemic conditions (hHCy)**. (+): increased number of cells and/or activity, (−): decreased number of cells and/or activity. Homocysteine induced neurotoxicity includes dysregulation in redox balance, lipoperoxidation and protein oxidation, Ca^2+^ pump dysfunction and activation of MAPKp38 which is detected in vulnerable cells by increased Fluoro-Jade C staining (+) and TUNEL positive cells (+). Ischemic preconditioning suppresses oxidative dysregulation and activates MAPK-ERK which leads to reduced Fluoro Jade C and TUNEL positivity in sensitive cells (Pavlikova et al., 2011; Petras et al., 2014; Kovalska et al., 2015; Lehotsky et al., 2015; Škovierová et al., 2015). Adapted from Lehotsky et al. (2015).

Taken together, documented responses of neuronal cells to hHcy, IRI and preischemic challenge in the hHcy model in rats (Figure 3) might suggest a correlation of several ethiological factors such as antioxidant defense (Borowczyk et al., 2012; Petras et al., 2014), alterations in the mechanisms of Ca^2+^ transport (Pavlíková et al., 2009) and likely newly explored epigenetic mechanisms, such as DNA methylation and chromatin remodeling in the phenomenon of ischemic damage and ischemic tolerance (Dirnagl et al., 2009; Lehotský et al., 2009a; Kalani et al., 2013; Thompson et al., 2013; Stetler et al., 2014).

## Conlusion and challenges

hHcy manifested as an elevated plasma level of Hcy is now a widely recognized risk factor for human vascular disorders and ischemic and tromboembolic stroke. It remains unclear whether excessive Hcy concentration directly contributes to the pathogenesis of the diseases or it represents a biomarker of metabolic aberrations, such as aberrant methyl group metabolism. Different strategies to reduce plasma Hcy concentrations have reached nonconclusive results, not just in the case of vascular disorders, but also with respect to neurodegenerative disorders, or cancer. Chronic elevated plasma hHcy alters functions of the vascular endothelial cells and, by the important pathobiochemical modifications, activates thiolation and homocysteinylation of plasma proteins and enzymes with the deleterious impact on the cerebrovascular permeability and eventually on brain parenchyma. Neural cells are sensitive to prolonged hHcy treatment, because Hcy cannot be metabolized by transsulfuration pathway either by folate or by vitamine B_12_ independent remethylation pathway. Therefore, Hcy could be used as an additional valuable prognostic and predictive biomarker in neurodegenerative diseases. Chronic challenge to hHCy induces posttranslational protein modifications which modifies the function and activity of the regulatory enzymes involved in free radical protection. Hcy as a toxic metabolic intermediate interferes with the proper redox balance and increases oxidative stress and production of free radicals in many cells including endothelial, glial and neuronal cells. Chronic elevated level of Hcy contributes to the increase of pathological neuronal lipoperoxidation and cellular protein oxidation, all the products being clearly recognized in the neurotoxicity and processes of brain damage. Moreover, Hcy treatment of cell cultures doubles the rate of telomere shortening (Zhang et al., 2016). Therefore, it would be very important to find the strategies to decrease Hcy levels. Genetic abnormalities and nutritional deficiencies explain only a part of hHcy pathologies. Hormonal and metabolic factors and also therapy with multiple vitamins and folate might be as a one clue to correct Hcy level in patients, however, clinical trials are needed to determine the optimal doses of vitamins.

The epigenetic mechanisms play an important role in elevated Hcy production. Since SAM is a universal donor of methyl group, SAH followed by Hcy are produced during these processes. It becomes more and more evident, that DNA methylation impairment might be a consequence of hHcy caused by endogenous (polymorphism of Hcy and folate pathways genes) and/or exogenous factors (dietary deficiency of folate and vitamins) and may be involved in hHcy pathogenesis. Thus, revealing many factors that may affect the methyl balance and understanding the pathophysiology of diseases from “methylation point of view” still remain a great challenge. Advanced studies are needed to understand whether and how changes in DNA methylation patterns, global and gene specific are associated with elevated levels of Hcy in the context to diseases and risk factors, such as oxidative stress, aging and exposure to drugs.

In this review we made an attempt to summarize and also emphasizes the results of novel experimental paradigm which combines only hHcy conditions (as a clinically recognized risk factor of ischemic stroke) and together with the ischemic insult and preconditioning maneuvers. Induction of hHCy alone leads to progressive neuronal cell death and morphological changes in the hippocampus and cerebral cortex in the rats. Ischemic reperfusion injury activates degeneration processes and de-regulates intracellular signaling which are aggravated under hHcy conditions.

The preischemic maneuver (preconditioning) evolves the state of ischemic tolerance manifested by the attenuated extent of neuronal degeneration as well as the intracellular signaling involved in the tolerance phenomenon. Combination of preconditioning with the hHcy retains the integrity of the majority of hippocampal neurons and also stimulates recovery of the expression rate of both the SPCA1 mRNA and the protein. Preconditioning even if combined with hHCy could still preserve the neuronal tissue from the lethal ischemic effect. The studies also emphasize the opposing effects of MAPK/ERK and MAPK/p38 signaling pathway on cell survival and cell death in the condition of preconditioned hHcy ischemia in the rat model of human stroke. The increased prevalence of hHcy in the population and crucial role of elevated Hcy levels in pathogenesis of different diseases, make this amino acid as an interesting target for future research.

In the human brain pathology, IPC is hardly suitable as a preventative measure in the predispose cerebrovascular patients. However, it could be used as a precaution against secondary stroke following medical procedures such as aneurysm repair or cardiac surgery. From the clinical point of view, use of preischemia/preconditioning challenge may bring some complications (Dirnagl et al., 2009; Lehotský et al., 2009a). Initial pre-clinical studies of this phenomenon revealed that pharmacological agents are also effective as preconditioners and there are also some other agents already clinically used. In the case of naturally occurring human strokes which cannot be predicted, maneuver of postconditioning may be a therapeutic strategy that could be used afterwards to elevate or stimulate repair mechanisms or as a precaution against stroke recurrence. It is also reasonable to summarize that preconditioning has a beneficial effect in the short term, however, the analysis of structural alterations in brain parenchyma documents that the damaging effect is only postponed. More clinically relevant experiments and clinical studies are urgently required to validate the efficacy of these paradigms in humans (Dirnagl et al., 2009).

Further investigations of the protective factors leading to ischemic tolerance and recognition of the co-morbid risk factors would result in development avenues for exploring novel therapeutics against ischemia and stroke. Usefulness and validity of Hcy as a biomarker of some diseases is another research challenge.

## Author contributions

All authors listed, have made substantial, direct and intellectual contribution to the work, and approved it for publication.

### Conflict of interest statement

The authors declare that the research was conducted in the absence of any commercial or financial relationships that could be construed as a potential conflict of interest.
